# COVID-19 Unveiling Heart Failure in the Realm of Rheumatic Heart Disease

**DOI:** 10.7759/cureus.52903

**Published:** 2024-01-25

**Authors:** Monika Karki, Pramod Bhattarai, Riya Mohan, Faraaz Mushtaq

**Affiliations:** 1 Cardiovascular Medicine, Broward Health, Fort Lauderdale, USA; 2 Pulmonary Medicine, Howard University Hospital, Washington, DC, USA; 3 Critical Care Medicine, Larkin Community Hospital Palm Springs Campus, Hialeah, USA; 4 Internal Medicine, Harlem Hospital Center, New York, USA; 5 Internal Medicine, University of Miami/John F. Kennedy (JFK) Medical Center, Atlantis, USA; 6 Cardiology, Broward Health Medical Center, Fort Lauderdale, USA

**Keywords:** mitral regurgitation, aortic regurgitation, aortic stenosis, tricuspid regurgitation, rheumatic mitral stenosis, systolic heart failure, atrial flutter with rapid ventricular response, covid-19, rheumatic heart disease

## Abstract

The global coronavirus disease 2019 (COVID-19) pandemic, caused by severe acute respiratory syndrome coronavirus 2 (SARS-CoV-2), has resulted in various clinical manifestations, including cardiovascular complications. This case report focuses on a unique instance where COVID-19 infection exacerbated heart failure and induced atrial fibrillation in a previously asymptomatic young male with undiagnosed rheumatic heart disease (RHD). RHD, a prevalent cause of valvular abnormalities in developing countries, poses an additional risk for severe outcomes when coexisting with COVID-19 infection, highlighting the need for prompt and tailored interventions.

## Introduction

First identified in Wuhan, China, in December 2019, severe acute respiratory syndrome coronavirus 2 (SARS-CoV-2) has rapidly evolved into a global pandemic, affecting more than nine million individuals worldwide [1]. Coronavirus disease 2019 (COVID-19) exhibits a diverse array of symptoms, commonly presenting with fever, respiratory distress, myalgia, weakness, and various cardiovascular complications such as stroke, infective endocarditis, heart failure, myocarditis, arrhythmias, myocardial infarction, and cardiogenic shock [2].

Rheumatic heart disease (RHD) remains a leading cause of valvular heart disease, causing substantial cardiovascular morbidity and mortality, especially among young individuals in developing nations, with approximately 319,400 deaths reported annually worldwide in 2015 [3,4]. Originating from valvular damage induced by an abnormal immune response to group A streptococcal infection, often contracted during childhood, RHD typically manifests as shortness of breath [5,6]. Left-sided heart involvement is more common in RHD, with the mitral valve being the most affected, followed by the aortic, tricuspid, and pulmonic valves [7,8]. Mitral regurgitation (MR), the most prevalent valvular lesion, may remain asymptomatic for several years due to compensatory left atrial and left ventricular dilatation [5]. Aortic regurgitation (AR) typically coexists with MR, while mitral stenosis (MS) tends to develop later in the disease course, and tricuspid regurgitation (TR) is often a consequence of MS, associated with elevated pulmonary pressure and ventricular dilatation [5]. Failure to institute timely intervention can lead to the progression of RHD to severe heart failure [5,8].

## Case presentation

**Figure 1:**
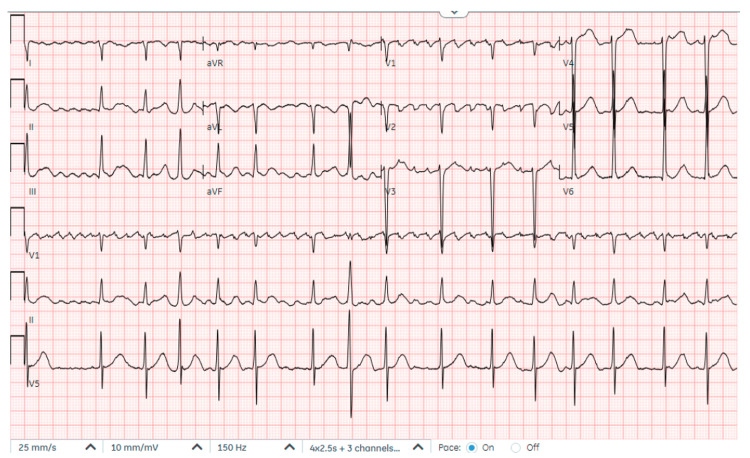
EKG showing atrial flutter with variable block

**Figure 2:**
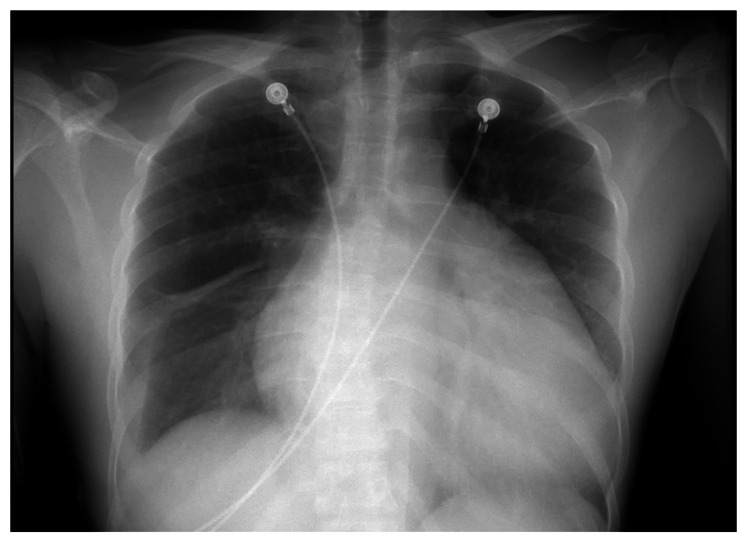
CXR showing cardiomegaly, vascular congestion, and small bilateral pleural effusion CXR: Chest X-ray

A 29-year-old male from Indonesia previously asymptomatic was transferred to the emergency department (ED) from a cruise ship for management of shortness of breath and atrial fibrillation. He had shortness of breath, pleuritic chest pain, and orthopnea for a week and was diagnosed with atrial fibrillation. He was treated with amiodarone, furosemide, and low-molecular-weight heparin (LMWH) on a cruise ship prior to transfer to the hospital. On arrival to the ED, he was afebrile, HR 112 bpm, BP 107/79 mm Hg, and saturating at 98% on room air. Physical examination revealed an irregularly irregular rhythm, palpable apical pulse, S3 gallop, rumbling diastolic murmur with diastolic snap in the left lateral position, decreased breath sounds in bilateral lungs, and trace pitting bilateral lower extremity edema. Laboratory findings showed white blood count 13.19 x10^3^/L, sodium 133 mmol/L, potassium 4.2 mmol/L, blood urea nitrogen (BUN) 22 mg/dL, creatinine 1.4 mg/dL, pro-B-type natriuretic peptide level 1250 pg/mL, troponin 0.04 ng/mL, thyroid-stimulating hormone (TSH) 1.14 mIU/mL, lactate dehydrogenase (LDH) 403 U/L, aspartate aminotransferase (AST) 75 U/L, and alanine transaminase (ALT) 65 U/L. The patient tested positive for COVID-19. EKG revealed atrial flutter with a variable block (Figure 1), and the chest X-ray showed cardiomegaly, vascular congestion, and small bilateral pleural effusion (Figure 2). He was initiated on antibiotics, amiodarone, intravenous furosemide, and anticoagulation with LMWH.

**Figure 3:**
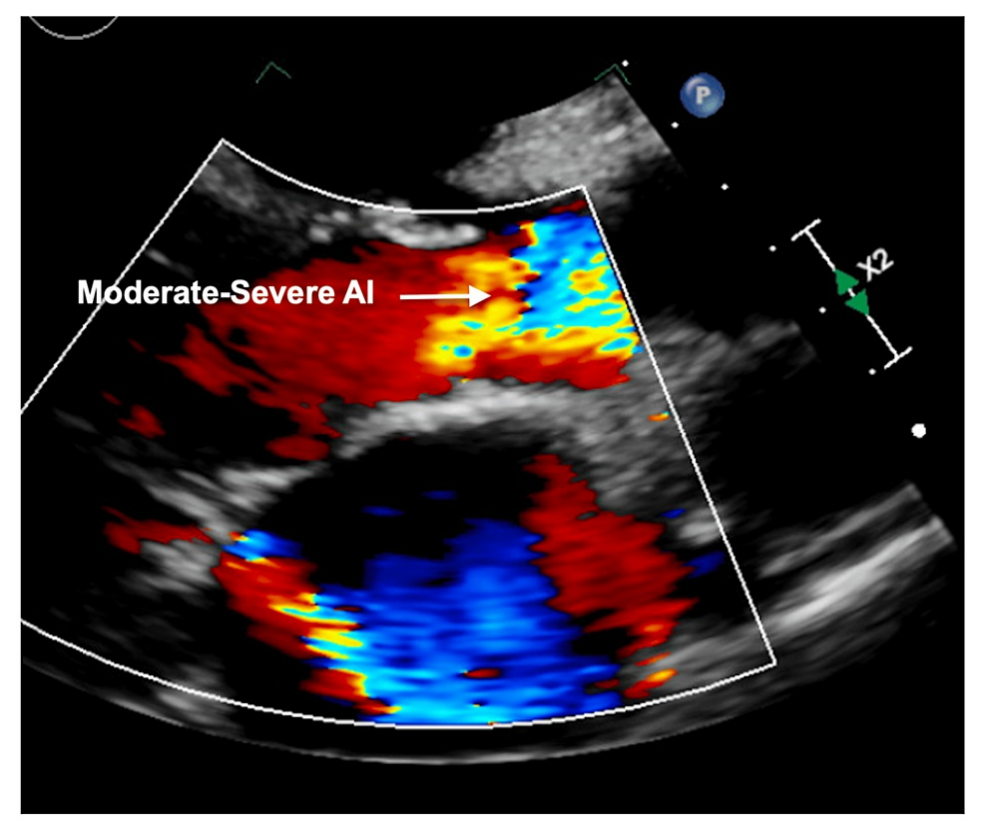
Transthoracic echocardiography parasternal long-axis view showing moderate to severe AR AR: Aortic regurgitation

**Figure 4:**
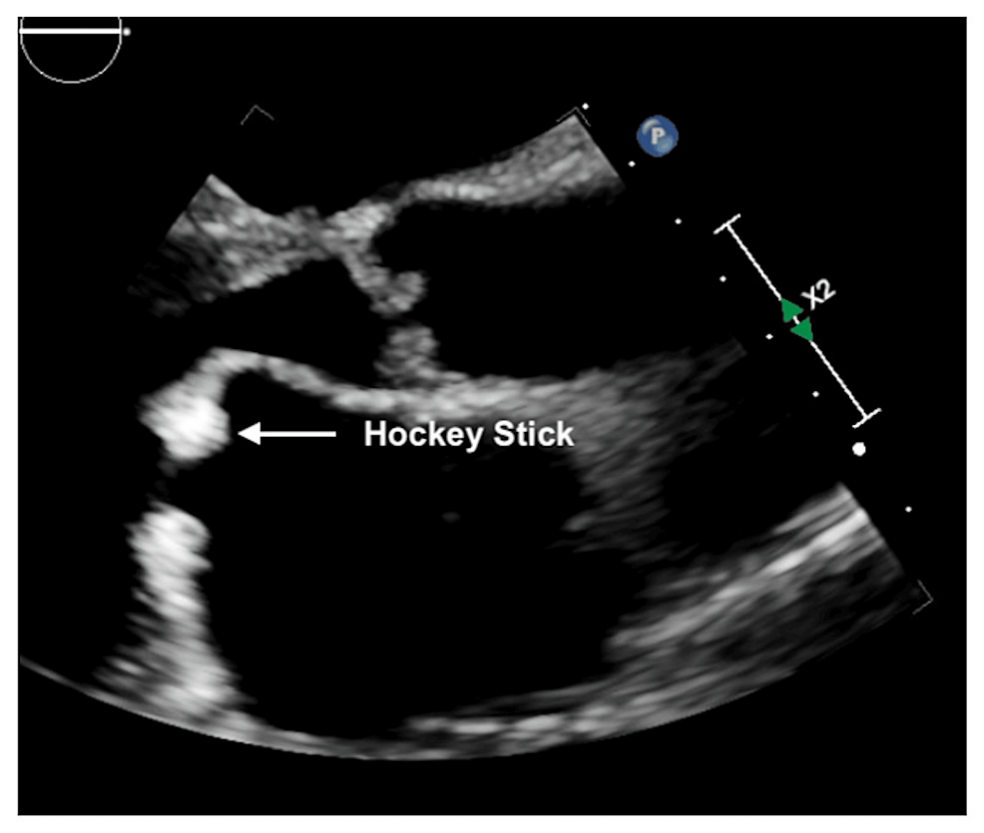
Transthoracic echocardiography parasternal long-axis view showing hockey stick of anterior and posterior mitral valve leaflets

**Figure 5:**
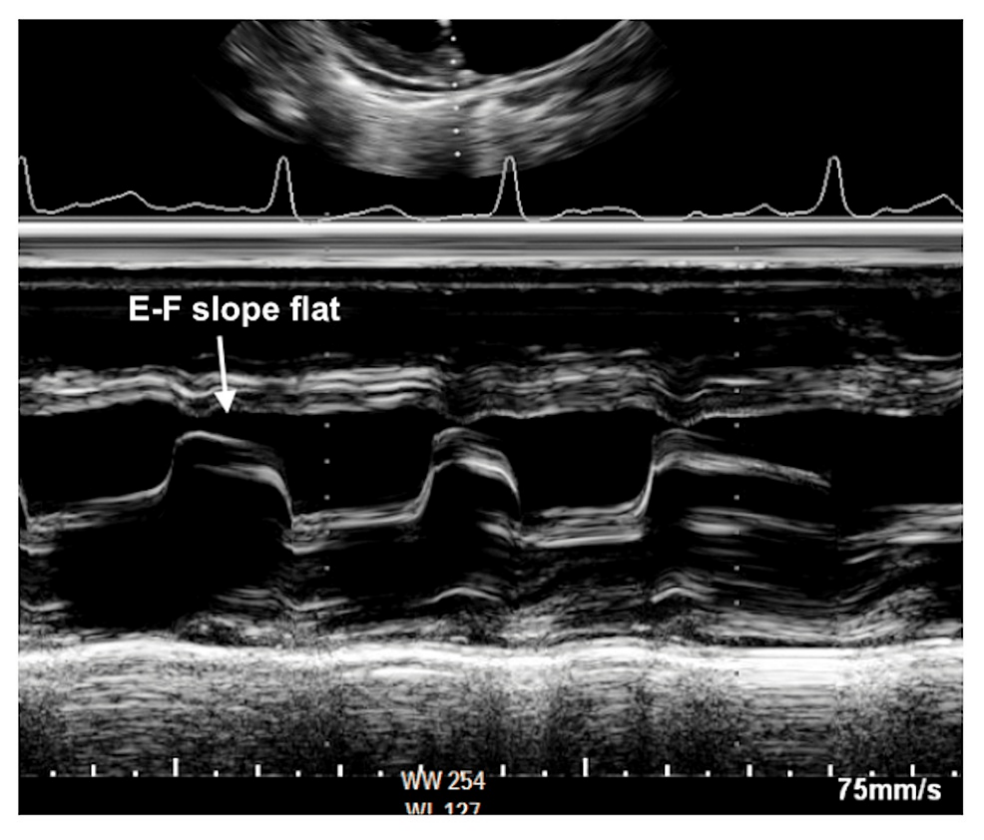
Transthoracic echocardiography M-mode showing E-F slope flattening

**Figure 6:**
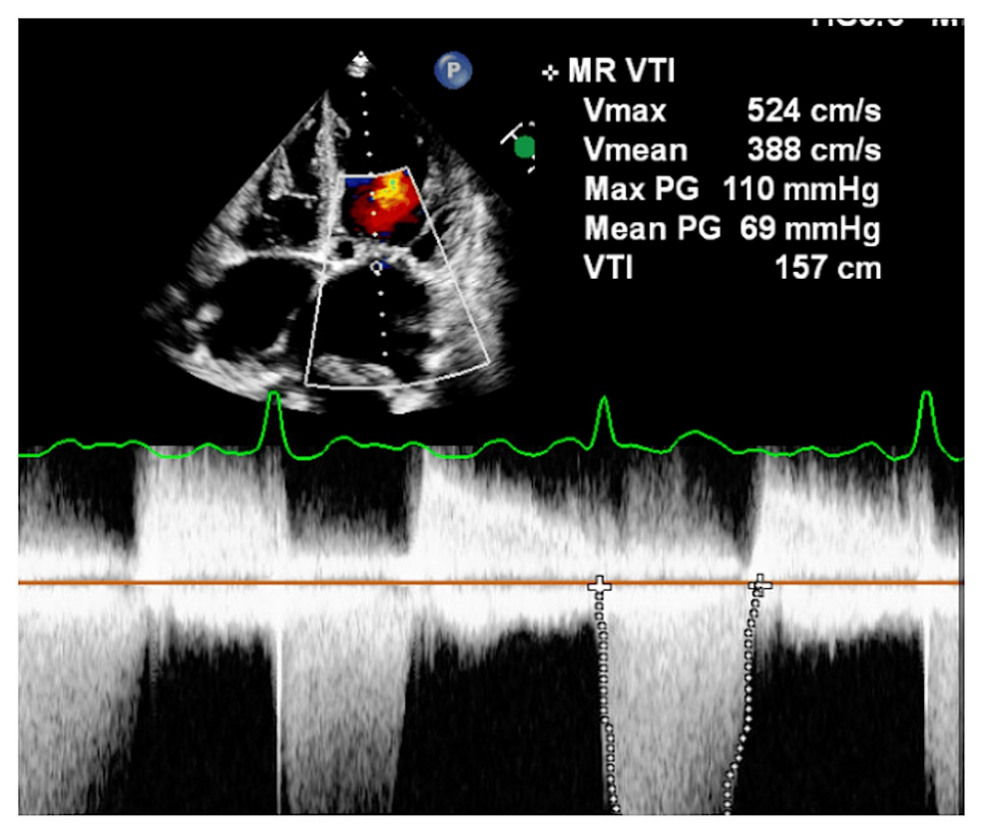
Transthoracic echocardiography continuous wave Doppler showing moderate MS MS: Mitral stenosis

**Figure 7:**
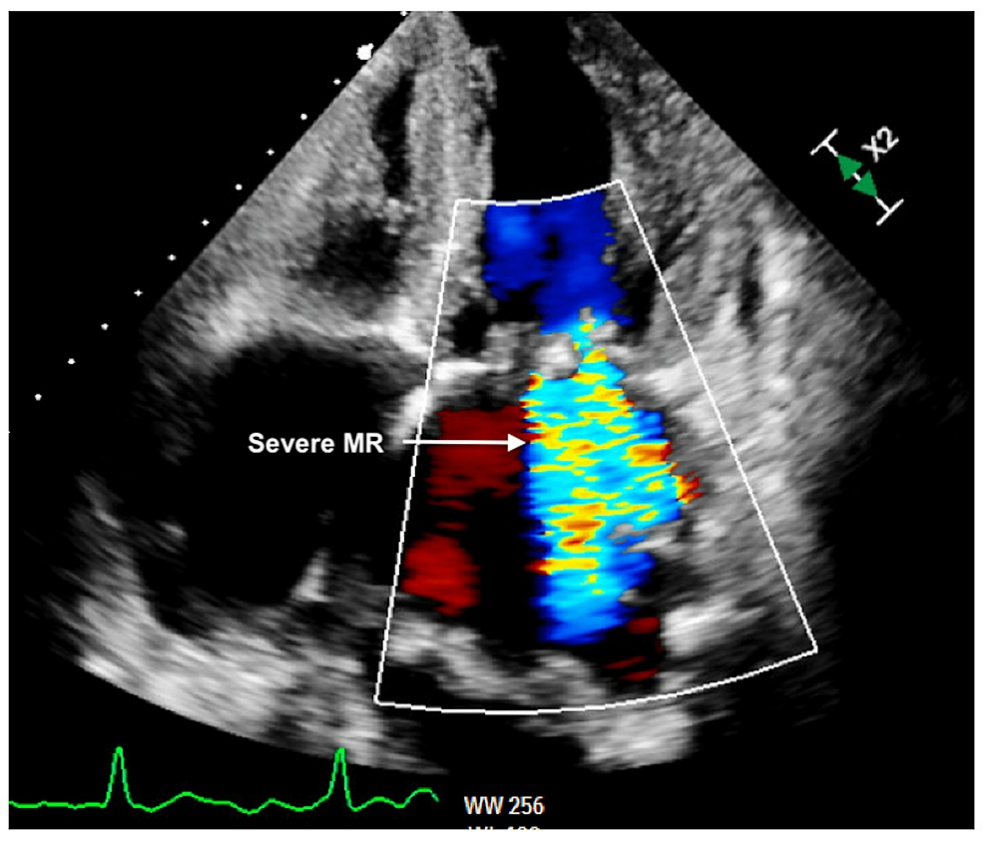
Transthoracic echocardiography four-chamber view showing severe MR MR: Mitral regurgitation

**Figure 8:**
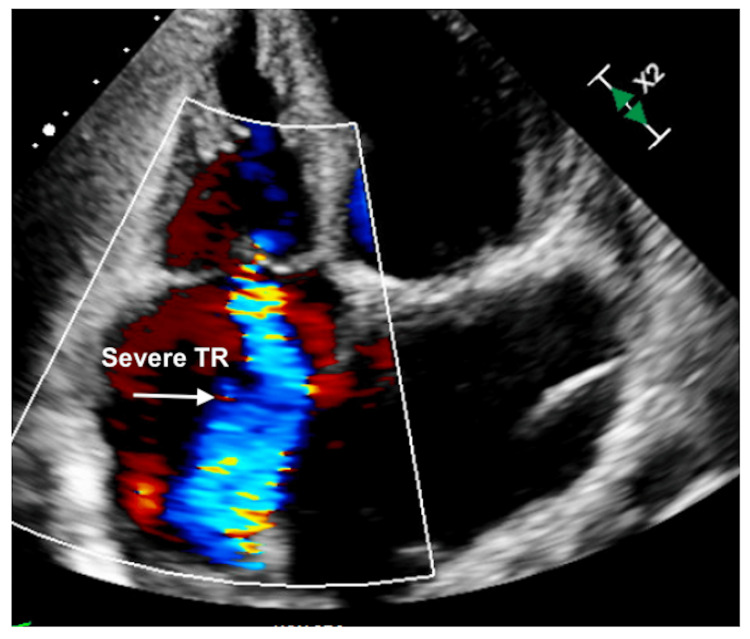
Transthoracic echocardiography four-chamber view showing severe TR TR: Tricuspid regurgitation

He was admitted to the telemetry floor for decompensated heart failure and arrhythmia management. Beta-blocker was held as he was in acute heart failure exacerbation and was loaded with digoxin. Echocardiography revealed left ventricular internal end-diastolic diameter (LVIDd) of 5 cm, moderately reduced left ventricular ejection fraction (LVEF) 40-45%, severely dilated left atrium, moderate-severely dilated right atrium, moderate-severe AR (Figure 3), mild-moderate aortic stenosis (AS), moderate MS with doming of anterior mitral leaflet (Figures 4-6), severe MR (Figure 7), and severe TR (Figure 8). After normalization of kidney function and optimal diuresis, he was initiated on goal-directed medical therapy (GDMT) for heart failure with metoprolol succinate, lisinopril, spironolactone, and dapagliflozin. Warfarin was initiated after bridging with LMWH for atrial flutter/fibrillation with underlying MS.

Cardiac catheterization was offered to evaluate his coronaries and surgical intervention for his multi-valvular disease was discussed. However, he refused cardiac catheterization and any invasive procedures. He opted for medical management and to follow up in his home country. He was discharged on the sixth day of hospitalization, with all his medical records, and was instructed to follow up closely with his primary care physician, cardiologist, and cardiothoracic surgeon. 

## Discussion

In the management of severe valvular disease associated with RHD, the preferred approach typically involves surgery or catheter-based procedures. However, in resource-constrained settings, medical management takes precedence. Diuretics play a crucial role in alleviating symptoms, particularly for patients with rheumatic tricuspid valve issues [4,6]. Beta-blockers are beneficial for isolated MS, enhancing diastolic filling through heart rate reduction [4]. In cases of severe mitral or aortic regurgitation, angiotensin-converting enzyme inhibitors or angiotensin receptor blockers are recommended to reduce afterload, while surgical intervention becomes imperative for severe AS [4].

Indications for surgery in RHD require a personalized approach, considering the patient’s condition, valve lesions, surgical capabilities, socio-economic factors, and individual patient preferences. Surgery is considered for symptomatic valve issues that are not manageable through catheter-based methods, particularly in cases of mixed, multivalve, or moderate-severe valvular heart disease [4,6,9]. Mitral valve repair is preferred for preserving ventricular function, while rheumatic aortic valves are typically replaced [3,4]. Percutaneous mitral balloon commissurotomy is the treatment of choice for isolated MS, although instances involving both MS and MR may require surgical replacement [3,4]. Atrial fibrillation or flutter in the context of MS in RHD warrants anticoagulation with warfarin [3,6]. Long-term follow-up involves penicillin prophylaxis, heart failure medications, and potential surgical or catheter-based interventions [6].

Our patient had atrial flutter/fibrillation and decompensated heart failure following COVID-19 infection, revealing previously undiagnosed RHD with multi-valvular disease. Subsequently, GDMT for heart failure management was initiated. Due to underlying multi-valvular pathology of moderate MS with severe MR and mild-moderate AS with moderate-severe AR, he did not qualify for valve repair. Based on his LVIDd, LVEF, and mitral and aortic valve pathologies, he needed valve replacement. Risk and benefits of surgical intervention were discussed; however, he opted for medical management and expressed to have surgery in his country.

## Conclusions

The presence of pre-existing cardiovascular disease has been identified as a significant risk factor for severe COVID-19 infection, yet the literature lacks a dedicated exploration of the intersection between valvular heart disease and COVID-19. This case highlights the heightened vulnerability and exacerbated outcomes associated with COVID-19 infection in individuals with previously undiagnosed multi-valvular RHD. The scarcity of reported cases emphasizes the need for further research and increased awareness concerning the impact of COVID-19 on individuals with diverse cardiovascular conditions, particularly those involving valvular pathology. A comprehensive understanding and recognition of these interactions are crucial for refining clinical management strategies and improving outcomes in patients concurrently facing valvular heart disease and COVID-19 infection. This case highlights the significance of embracing a comprehensive and individualized treatment approach in managing complex cases while respecting patient preferences.
